# Development of salinity tolerance in rice by constitutive-overexpression of genes involved in the regulation of programmed cell death

**DOI:** 10.3389/fpls.2015.00175

**Published:** 2015-03-30

**Authors:** Thi M. L. Hoang, Lalehvash Moghaddam, Brett Williams, Harjeet Khanna, James Dale, Sagadevan G. Mundree

**Affiliations:** ^1^Centre for Tropical Crops and Biocommodities, Queensland University of TechnologyBrisbane, QLD, Australia; ^2^Sugar Research AustraliaBrisbane, QLD, Australia

**Keywords:** anti-apoptotic, programmed cell death, TUNEL, ROS, salinity stress, rice, abiotic stress, apoptosis

## Abstract

Environmental factors contribute to over 70% of crop yield losses worldwide. Of these drought and salinity are the most significant causes of crop yield reduction. Rice is an important staple crop that feeds more than half of the world’s population. However among the agronomically important cereals rice is the most sensitive to salinity. In the present study we show that exogenous expression of anti-apoptotic genes from diverse origins, *AtBAG4 (Arabidopsis)*, *Hsp70 (Citrus tristeza virus)* and *p35* (Baculovirus), significantly improves salinity tolerance in rice at the whole plant level. Physiological, biochemical and agronomical analyses of transgenic rice expressing each of the anti-apoptotic genes subjected to salinity treatment demonstrated traits associated with tolerant varieties including, improved photosynthesis, membrane integrity, ion and ROS maintenance systems, growth rate, and yield components. Moreover, FTIR analysis showed that the chemical composition of salinity-treated transgenic plants is reminiscent of non-treated, unstressed controls. In contrast, wild type and vector control plants displayed hallmark features of stress, including pectin degradation upon subjection to salinity treatment. Interestingly, despite their diverse origins, transgenic plants expressing the anti-apoptotic genes assessed in this study displayed similar physiological and biochemical characteristics during salinity treatment thus providing further evidence that cell death pathways are conserved across broad evolutionary kingdoms. Our results reveal that anti-apoptotic genes facilitate maintenance of metabolic activity at the whole plant level to create favorable conditions for cellular survival. It is these conditions that are crucial and conducive to the plants ability to tolerate/adapt to extreme environments.

## Introduction

By 2050, the world population is expected to reach 9.6 billion people (UNFPA, 2014). To sustainably provide sufficient food for the increasing population crop productivity needs to increase by ~44 million metric tons annually. This is a challenge because there is very little potential for future expansion of arable lands whilst climate predictions suggest that a larger portion of the globe will be subjected to erratic environmental conditions and abiotic stress (Eckardt, 2009; FAO, 2009, 2012; Cominelli et al., 2013). Two abiotic stress factors that significantly hinder world crop production are soil water deficit and salinization (Munns, 2011).

Rice (*Oryza sativa* L.) is an important crop that feeds more than half of the world’s population and is the model system for monocotyledonous plants that include members of the agronomically important cereals. Approximately 90% of the world’s production and consumption of rice are in Asia (Khush, 2005). Rice has been considered as the single most important source of employment and income for rural people in humid and sub-humid Asia, it provides 50–80% of the calories consumed (Hossain and Fischer, 1995; Khush, 2005).However, rice is very sensitive to salinity stress and is currently listed as the most salt sensitive cereal crop with a threshold of 3 dSm^-1^ for most cultivated varieties (USDA, 2013). Rice yield in salt-affected land is significantly reduced with an estimation of 30–50% yield losses annually (Eynard et al., 2005). Further yield losses due to climate change are predicted (Eynard et al., 2005).

Methods for salinity tolerance screening are important for the success of a breeding program. As improving yield of plants undergoing salinity stress is one of the main targets of plant breeding, salinity tolerance screening based on agronomical parameters such as growth, yield and yield components has become the method of choice by labs worldwide (Gregorio et al., 1997; Zeng et al., 2002; Lee et al., 2003; Moradi and Ismail, 2007; Cha-Um et al., 2009; El-Hendawy et al., 2009). Recently physiological parameters have also gained recognition as important selection criteria for screening salinity tolerance in plants due to the reliability of information attained (Ashraf, 2004; Munns et al., 2006; El-Hendawy et al., 2009).

To date, salinity tolerance strategies have utilized three major approaches: (i) conventional breeding, (ii) marker assisted selection and (iii) genetic engineering. Of these, genetic engineering displays great potential and has become a powerful tool in plant breeding programs since it allows the introduction of select gene(s) without affecting the desirable characteristics of an elite genotype (Bhatnagar-Mathur et al., 2008).Genetic engineering for salinity tolerance in plants has focused on genes that encode compatible organic solutes, antioxidants [detoxification of reactive oxygen species (ROS)], ion transport, heat-shock and late embryogenesis abundant proteins (Ashraf et al., 2008). Despite some promising reports, the development of cultivars with enhanced salinity tolerance using a transgenic approach is awaiting further investigation. Currently we are able to produce crops with enhanced salinity tolerance that survive in the glasshouse, however, once applied in the field the tolerance fails due to combined stresses; salinity is commonly associated with drought or temperature stress. One approach with prospective application for the generation of the “next frontier of crop plants” with broad-spectrum tolerance is the exogenous expression of anti-apoptotic genes that suppress innate programmed cell death (PCD) pathways.

Programmed cell death or simply “the decision of whether a given cell should live or die” is essential for all multicellular (Metazoan) organisms (Williams and Dickman, 2008). Under several stimuli, this decision is dependent on the battle between anti-apoptotic and pro-apoptotic (pro-death) proteins and signal transduction pathways (Li and Dickman, 2004; Williams and Dickman, 2008; Williams et al., 2014). Previous studies have assessed the applicability of anti-apoptotic genes for “broad stress tolerance,” however, these have focused primarily on model crops (Dickman et al., 2001; Doukhanina et al., 2006; Wang et al., 2009).

*AtBAG4, Hsp70* and *p35* are anti-apoptotic genes that have been reported to confer tolerance to salinity and drought stresses in transgenic tobacco. *AtBAG4* is a Bcl-2- associated athanogene from *Arabidopsis thaliana.* The *A. thaliana* genome contains seven homologs of the BAG family, including four with a domain organization similar to animal BAGs (Kabbage and Dickman, 2008). The BAG gene family has been identified in yeast, plants and animals, and is believed to function through a complex interaction with signaling molecules and molecular chaperones such as heat shock proteins (Hsp; Sondermann et al., 2001; Takayama and Reed, 2001; Doukhanina et al., 2006).

Heat shock proteins are powerful chaperones that are expressed in response to a variety of physiological and environmental stresses and are localized in a variety of sub-cellular organelles. By protein-protein interaction Hsp70 proteins can facilitate anti-apoptotic Bcl-2 proteins to inhibit apoptosis pathways at distinct key points (Joly et al., 2010). The broad-spectrum activity of Hsp70s requires the recruitment of co-chaperones and other chaperone systems (Brodsky and Bracher, 2007). Overexpression of *Hsp70* in tobacco conferred tolerance to drought stress (Cho and Hong, 2006).

The function of p35, a Baculovirus anti-apoptotic protein has been extensively studied in different organisms (Clem et al., 1991; Clem and Miller, 1994; Bump et al., 1995; Xue and Horvitz, 1995; Bertin et al., 1996). Expression of *p35* in tobacco, tomato and passion fruit significantly improved tolerance to abiotic and biotic stresses (Lincoln et al., 2002; Freitas et al., 2007; Wang et al., 2009). Transgenic tobacco expressing *p35* displayed broad-spectrum tolerance to a range of abiotic stresses including salinity, upon further investigation this tolerance was attributed to the ability to sequester ROS of p35 (Wang et al., 2009).

In plants, ROS are versatile molecules playing dual roles as both toxic compounds and signal transduction molecules that mediate responses to environmental stresses, pathogen infection, developmental stimuli and even PCD (Mittler et al., 2004, Miller et al., 2010). Accordingly, responses of plants to ROS are dose dependent, high ROS concentrations may cause cellular damage or trigger PCD while low ROS concentrations serves as signals for development and/or stress responses (Bhattacharjee, 2012).

Here we present for the first time the development of salinity tolerance in one of the most important staple crops – rice (*O. sativa* L.) by constitutive-overexpression of *AtBAG4* (NCBI Reference Sequence: NM_115037), *Hsp70* (GenBank: EU857538) and *p35* (GenBank: KF022001) and evaluation of their ability to confer salinity tolerance in rice using agronomical, biochemical and physiological assessments. By investigating the ability of anti-apoptotic genes (from different sources) to confer tolerance to salinity stress via maintenance of ROS, photosynthetic efficiency and growth rate thereby minimizing yield loss, a possible mechanism of anti-apoptotic genes to maintain metabolic activity in plants under salinity stress is also elucidated.

## Materials and Methods

### Generation of Constructs

*AtBAG4* from *A. thaliana*, *Hsp70* from *Citrus tristeza* virus and *p35* from Baculovirus driven by the maize polyubiquitin-1 (Ubi-1) promoter were cloned into the binary vector pCAMBIA1301. All constructs were sequenced prior to the transformation for confirmation of fidelity.

### Rice Transformation and Molecular Characterization of Transgenic Plants

Embryogenic calli were initiated from mature seeds of *O. sativa* L. ssp. Japonica cv. Nipponbare, and transformed by *Agrobacterium-*mediated transformation as described in Hoang (2014). Briefly, 300–320 clumps of embryogenic rice calli were co-cultivated with recombinant *Agrobacterium tumefaciens* (strain AGL1) harboring the respective genes of interest at 25–27°C in dark for 3 days. Following co-cultivation contaminating *Agrobacteria* were removed by washing in 2N6 liquid media and sterile water containing 200 mg/L^-1^ timentin before blotting dry on sterile Whatman^®^ filter paper and cultivation on 2N6 selection media supplemented with 200 mg/L^-1^ timentin and 25 mg/L^-1^ hygromycin; calli were transferred onto fresh 2N6 selection media containing 50 mg/L^-1^ hygromycin and 200 mg/L^-1^ timentin every 14 days. Seven weeks post-co-cultivation, individual proliferating callus clumps (cream white in color) were transferred to regeneration media supplemented with 200 mg/L^-1^ timentin and 25mg/L^-1^ hygromycin and incubated at 27°C in the dark for the first 7 days and then exposed to day/night cycle of 16/8 h at 25°C for shoot formation. Shoots with at least three well-formed leaves were transferred to rooting media supplemented with 200 mg/L^-1^timentin and 25 mg/L^-1^ hygromycin. Culture vessels were incubated in tissue culture growth room at 25°C with a 16 h photoperiod and monitored for plant growth, elongation and root development.

One plant from each petri dish was sampled for PCR and RT-PCR using gene specific primers and hygromycin specific primers (Supplementary **Table S1**). Briefly, genomic DNA was isolated from 100 mg of fresh leaf tissue using a DNeasy^®^ Plant Mini Kit (Qiagen, Valencia, CA, USA). PCR analysis was carried out in a 20 μL reaction mixture containing 2X GoTaq green (Promega, Madison, WI, USA), 5 pM each forward and reverse primers, 100 ng of genomic DNA and a volume of DNase, RNase free water up to 20 μL using standard PCR parameters. For RT-PCR, total RNA was isolated from 50 mg of leaf tissue using an RNeasy Plant Mini kit (Qiagen, Valencia, CA, USA) following the manufacturer’s instructions and treated with RNase free DNase (Promega, Madison, WI, USA) RT-PCR was carried out using the SuperScript^®^ III first strand synthesis system (Invitrogen-Life Technologies Australia Pty Ltd, Musgrave, VIC, Australia) using gene specific primers following the manufacturer’s instructions. Plants that were confirmed transgenic by PCR and having the gene of interest expressed (confirmed by RT-PCR) was considered as one transgenic line. This plant was multiplied *in vitro* until at least 30 clones (plants) were obtained.

### Salinity Stress Experiments at Seedling and Reproductive Stages

Rice plants were acclimatized from tissue culture in the glasshouse at 28/21°C day/night as described in Hoang (2014). Briefly, pots containing potting mix and rice plants were placed in a container filled with tap water for 14 days and Aquasol fertilizer (Yates, NSW Australia) was applied. One week post-fertilization the salinity stress experiment at seedling stage (three fully expanded leaves and the fourth leaf has emerged) was started; water in the container was removed and tap water supplemented with 100 mM NaCl was added until the water level reached ~1 cm above the level of soil. The water level was maintained daily at 1 cm above the soil level by adding tap water (not salt water) into the container. The salinity stress experiment at reproductive stage was carried out on 30 day-old acclimatized rice plants as described by Moradi and Ismail (2007) and Hoang (2014).

### Detection of H_2_O_2_

*In Situ* H_2_O_2_ production in rice leaves exposed to 100 mM NaCl for 30 h was detected by 3,3′-Diaminobenzidine (DAB) Enhanced Liquid Substrate System for Immuno-histochemistry solution, D3939 (Sigma, Saint Louis, MO, USA) following the manufacturer’s instructions. Briefly, pieces of the youngest fully expanded leaf (~1 cm long) were excised from rice plants and immediately immerging into 0.5 mL of mixed D3939 solution for 60 min at room temperature. The DAB solution was decanted and replaced with 1 ml of Ethanol: Acetic acid (3:1 v/v) for destaining of chlorophyll.

### TUNEL Assay

The Terminal deoxynucleotidyl transferased UTPNick End Labeling (TUNEL) assay was carried out using an *In Situ* Cell Death Detection Kit, Fluorescein (Roche Diagnostics Australia Pty Ltd, Castle Hill, NSW, Australia) following the manufacturer’s instructions. Briefly, root tips fragments (~1 cm) were taken from plants, washed three times with fresh phosphate buffer saline (PBS) and fixed in 4% paraformaldehyde solution at 4°C for 1 h. Once fixed, root tips were washed two times with fresh PBS before immersing in fresh permeability solution (0.1% triton X 100 and 0.1% sodium citrate) and microwaving at 700 W for 1 min. Fresh PBS were used to immediately cool the sample after microwaving, followed by two more PBS washes. The samples were then mixed with a 50 μL aliquot of TUNEL reaction mix in a 1.5 mL Eppendorf tube. As a negative control, a 50 μL aliquot of TUNEL label solution without enzyme was also included. Samples were incubated at 37°C for 1 h under high humidity, washed two times with fresh PBS and counterstained with 0.5 mg/mL propidium iodide (Sigma-Aldrich Pty. Ltd, Sydney, NSW, Australia) for 15 min in the dark at room temperature. Stained root tips were washed two times with fresh PBS and squash mounted onto slides and examined under a Nikon A1 confocal microscope.

### Electrolyte Leakage Measurement

Electrolyte leakage from rice leaves at the seedling and reproductive stages was measured using a CM 100-2 conductivity meter (Reid & Associates CC, South Africa). Briefly, detached leaves were sectioned into 0.5 cm pieces, washed two times with deionised water, blotted dry with paper towel and loaded into wells of the CM 100-2 conductivity meter containing 1.25 mL of deionised water. Measurements were taken at 2 min intervals over a 60 min period. Dry weight (DW) was determined after the samples were dried overnight in an oven at 70°C. Electrolyte leakage was normalized by DW and was calculated as the slope of electrolyte leakage over time.

### Infrared Spectroscopy

Fourier transform infrared (FTIR) was used to analyze the chemical composition of wild type (WT), vector control (VC), *AtBAG4, Hsp70* and *p35* leaves exposed to 0 and 100 mM NaCl. For analysis, 2 cm leaf sections excised 5 cm from the base of the flag leaf, were lysophilised in 2 mL Eppendorf tubes overnight at -83°C using a Flexi-Dry MPTm Freeze Dryer (FTS systems, Stone Ridge, NY, USA) under a vacuum pressure of <500 Kpa. Once freeze dried, samples were ground to a fine powder using a Qiagen Tissue Lyser and analyzed by ATR/FTIR spectroscopy. IR spectra were collected in the range of 4000 to 525 cm^-1^ using a Nicolet 870 Nexus FTIR spectrometer equipped with a smart endurance single bounce diamond ATR accessory (Thermo-Nicolet, Madison, WI, USA).

### Relative Water Content Determination

Leaf relative water content (RWC) was calculated using the method described by Lafitte (2002). A piece of leaf tissue (~10 cm) from the middle section of the youngest fully expanded leaf was excised, weighed [fresh weight (FW)] and placed in a 15 mL Falcon tube containing distilled water and kept in dark at 4°C overnight for determination of turgid weight (TW). To avoid error due to residual water prior to weighing, the leaves were blotted dry with tissue paper. For determination of DW samples were weighed after drying at 70°C in a vacuum oven for 3 days. The RWC was calculated as RWC = ((FW-DW)/(TW-DW))*100.

### Gas Exchange Measurements

Net photosynthesis (A) was measured using a LiCOR Infra-Red Gas Analyser LI-6400 XT (John Morris Scientific, Chatwood, NSW, Australia). Measurement of net photosynthesis at seedling stage was performed on the third leaf and was recorded at 0, 3, 7, 10, and 13 days after salinity treatment; reproductive stage measurements were taken on the flag leaf.

### Measurement of Leaf Sodium/Potassium Contents

The amount of Na^+^ and K^+^ in the leaf tissue of salinity-stressed plants at both the seedling and reproductive stages were determined using an Atomic Absorption Spectrophotometer (Shimadzu A-7000, Shimadzu Scientific Instrument, Sydney, NSW, Australia). Fifteen milligrams of dried leaf was cut into 0.5 cm long segments and immersed in 30 mL deionised water in a 50 mL Falcon tube as described in Dionisio-Sese and Tobita (2000). The mixture was boiled in a water bath for 1 h followed by 20 min autoclaving at 121°C. Samples were cooled down to room temperature and filtered using the Whatman filter paper No 40 (Thomas Scientific, Swedesboro, NJ, USA).

### Statistical Analysis

All experiments in this study were conducted using a randomized block design and where applicable, data were analyzed using one-way ANOVA and Tukey’s HSD (honest significant difference) or Fisher’s LSD (least significant difference) tests (Minitab Version 16).

## Results

### Generation of Transgenic Rice Expressing Anti-Apoptotic Genes

The TUNEL assay confirmed that salinity induces PCD in WT Nipponbare rice (**Figure S1**). To study the potential of the manipulation of PCD pathways for enhancing tolerance to salinity stress in rice, the coding regions of the *AtBAG4*, *Hsp70* and *p35* genes were placed under the control of the maize ubiquitin-1 promoter (**Figure S2A**). The respective expression cassettes and a VC (the vector backbone without gene of interest, VC) were introduced to *O. sativa* L. ssp. japonica cv. Nipponbare by *Agrobacterium*-mediated transformation of Nipponbare embryogenic calli. Fifty-six PCR-confirmed transgenic lines were generated (**Figure S2B**). The expression of *AtBAG4*, *Hsp70* and *p35* in the transgenic lines was examined using RT-PCR (**Figure S2C**). The RT-PCR results show that 100% of PCR-positive transgenic lines of AtBAG4 and Hsp70 have the gene of interest expressing and approximately 90% of PCR-positive lines expressing p35 (Supplementary **Table S1**).

The WT, VC and the transgenic plants expressing anti-apoptotic genes were grown under normal growth conditions to reveal any differences in morphology and physiology between the plants at the seedling and reproductive stages. The results show that no significant difference was observed between WT, VC and the transgenic plants under normal growth conditions at both developmental stages (**Figure S3** and **Figure S4**).

An initial screening for salinity stress tolerance was conducted on 10 transgenic rice lines (10 plants/line) containing each of *AtBAG4*, *Hsp70* and *p35*, VC expression cassettes and WT Nipponbare in a glasshouse at seedling stage (≈ 2 weeks post-acclimation). Morphological data such as leaf damage and survival rate were captured and analyzed (**Figure S3**). The results showed that more than 70% of the transgenic lines tested exhibited higher survival levels and less leaf damage in comparison to the WT and VC plants (**Figure S5**).The best transgenic lines expressing each of the above genes were selected for further study.

### Expression of Anti-Apoptotic Genes Suppresses Salinity-Induced Programmed Cell Death and Promotes Reduced Cellular ROS Levels During Salinity Stress

**FIGURE 1:**
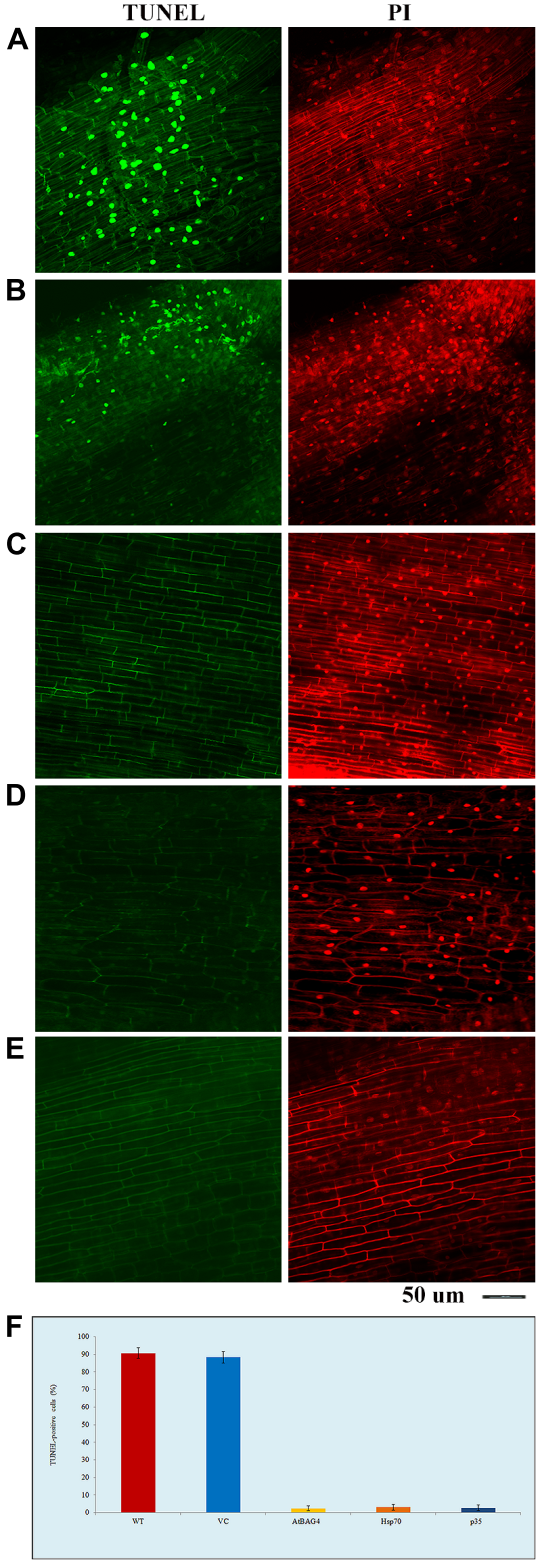
**Transgenic rice expressing *AtBAG4*, *Hsp70* and *p35* can reduce cell death under salinity stress. (A)** Wild type (WT); **(B)** Vector-control (VC); **(C)** *AtBAG4*; **(D)** *Hsp70*; and **(E)** *p35* root tips, **(F)** TUNEL-positive cells. WT, VC, *AtBAG4*, *Hsp70* and *p35*transgenic plants were subjected to 100 mM NaCl, TUNEL assay and propidium iodide counter-staining were carried out at 36 h after salinity stress. Nucleic acid in TUNEL positive cells are selectively stained and fluoresces green, indicating the presence of apoptotic-like bodies, whereas all nucleic acid is counter- stained with propidium iodide and fluoresces red. Magnifications as indicated.

To examine whether expression of anti-apoptotic genes prevents salinity-induced PCD, TUNEL assays were conducted on root tips of rice plants expressing *AtBAG4, Hsp70* and *p35* after 36 h exposure to 100 mM NaCl; WT and VC plants were also included as controls. The data show that there was no or very little cell death in transgenic rice plants expressing anti-apoptotic genes while noticeable cell death was observed in WT and VC plants (**Figure 1**). As expected, no cell death observed in the non-stressed WT, VC or transgenic controls (data not shown).

**FIGURE 2:**
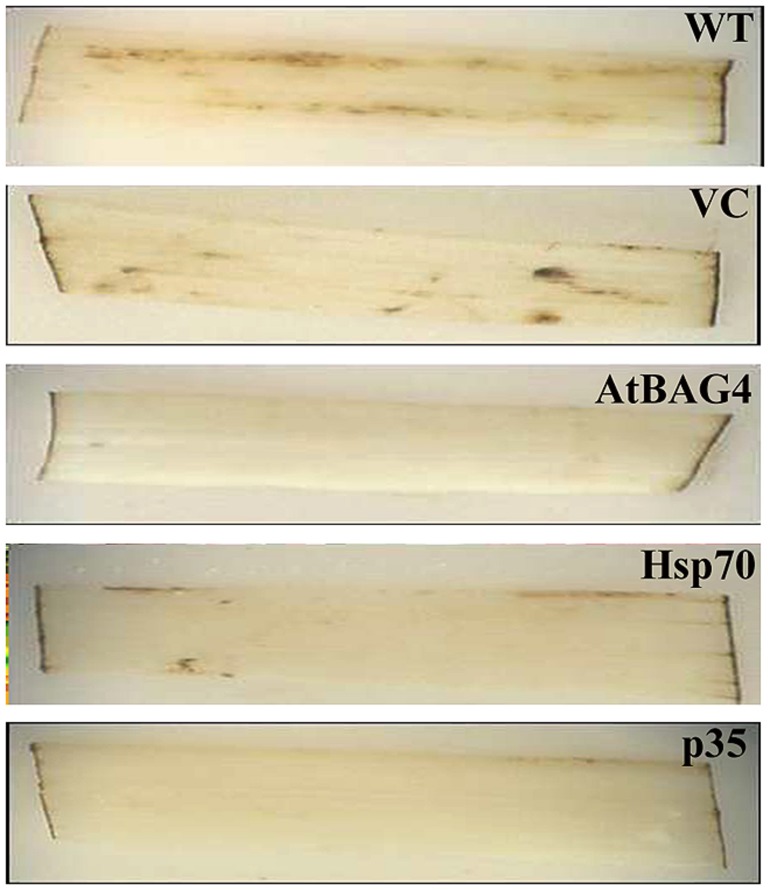
**Expression of anti-apoptotic gene suppresses ROS (H_2_O_2_) level in leaf of rice exposed to 100 mM NaCl**. DAB (3,3′-DAB) staining was conducted 30 h after salinity stress.

Programmed cell death has shown to be triggered by signals including ROS that originate from different organelles such as the chloroplast and mitochondria (Foyer and Noctor, 2005; Rhoads et al., 2006). ROS levels were reported to increase in plant cells during salinity stress (Borsani et al., 2005; Zhu et al., 2007; Chawla et al., 2013). To elucidate whether the expression of anti-apoptotic genes in rice coincides with reduced ROS production caused by salinity stress rice leaves were assessed for *in situ* H_2_O_2_ production by DAB staining 30 h post exposure to 100 mM NaCl. As shown in **Figure 2** more H_2_O_2_ production was observed in WT and VC leaves while lower levels of H_2_O_2_ were detected in the leaves of transgenic plants expressing the anti-apoptotic genes. This result indicates that the expression of anti-apoptotic genes is associated with reduced H_2_O_2_ production during salinity stress.

### Expression of Anti-Apoptotic Genes Prevents Changes in Cellular Chemical Composition During Salinity Stress

**FIGURE 3:**
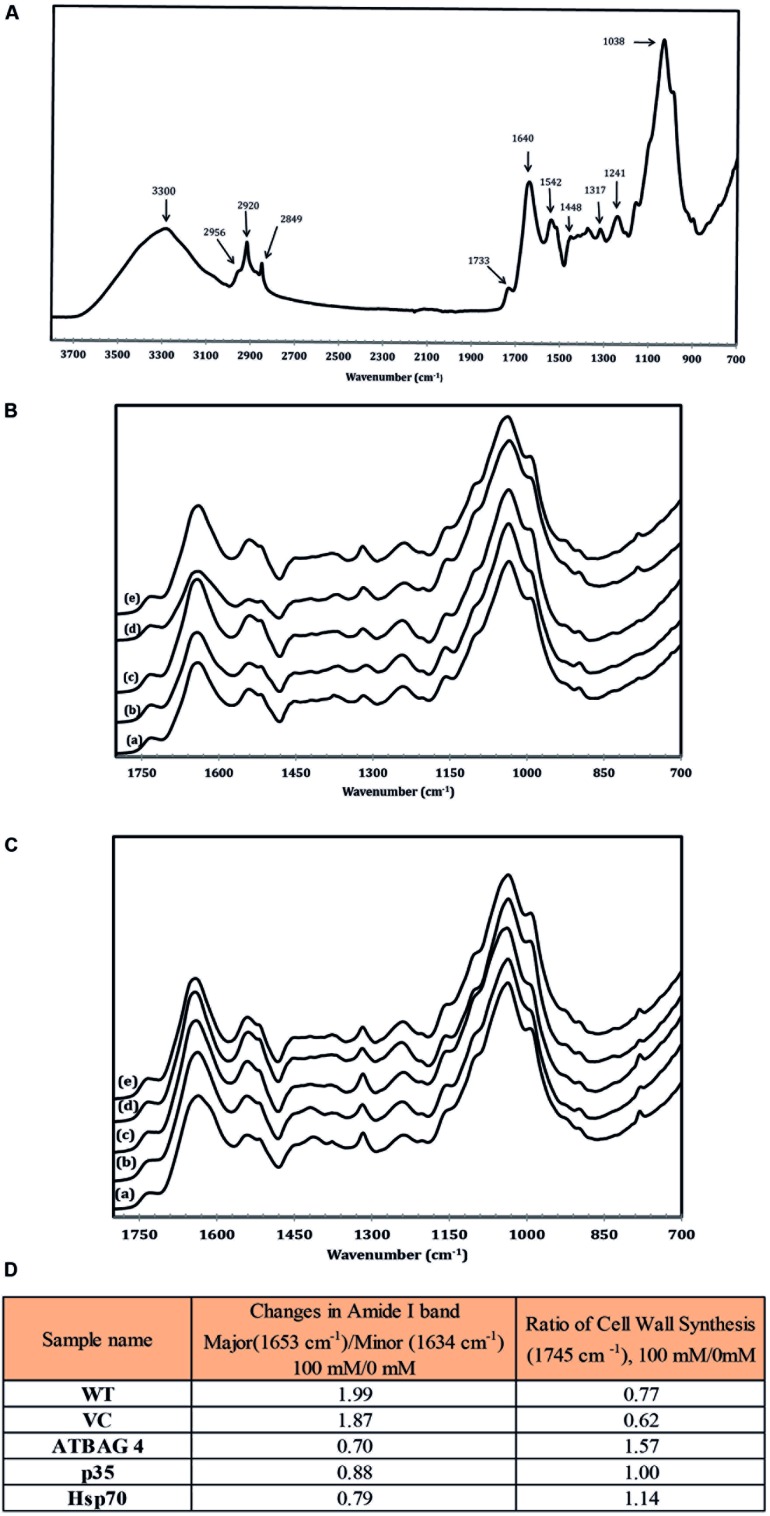
**(A)** IR spectroscopic characteristic of wild type rice in non-stress conditions. **(B)** IR spectroscopic characteristic of (a) WT, (b) VC, (c) *p35*, (d) *Hsp70* and (e) *AtBAG4* leaf rice samples in non-stress conditions.**(C)** IR spectroscopic characteristic of (a) WT, (b) VC, (c) *p35*, (d*) Hsp70* and (e) *AtBAG4* leaf rice samples at100 mM NaCl. **(D)** FTIR analysis of protein structure and cell wall synthesis.

Fourier transform infrared spectroscopy, FTIR, is an established and powerful technique for the analysis of structural and composition changes in both animal and plant cells (Griffiths, 1975; Ellerbrock et al., 1999; Barth, 2000; Yang and Yen, 2002; Meissl et al., 2007). To investigate potential protein structural and chemical compositional changes that occur during salt stress IR spectra were obtained and analyzed from rice leaves harvested from plants treated with 0 mM and 100 mM NaCl. Previously, studies have established the deconvolution and analysis of two absorption regions as key indicators of stress levels and metabolic status, amide I (1580–1700 cm^-1^), and pectin accumulation (1745 cm^-1^; Griffiths, 1975; Barth, 2000; Yang and Yen, 2002); we therefore focused on these spectral regions. Moreover, the amide I absorption region is particularly sensitive to salt stress and proteins with higher proportions of the minor amide I band (1633 cm^-1^) rather than the major band (1653 cm^-1^) have been associated with salinity tolerance and protein stability in the ice plant (*Mesembryanthemum crystallinum*) due to more ordered hydrogen bonding between the peptide bonds (Yang and Yen, 2002). Therefore, protein stability can be accurately assessed by measuring the ratio of the major and minor bands of non-stressed and stressed samples. As can be seen in **Figure 3**, the ratio of the major band of the WT and transgenic control samples increased substantially (>1.5) upon comparison of the 0–100 mM treated samples. In contrast, transgenic lines expressing *AtBAG4* and *Hsp70* maintained a higher proportion of the minor band and gave low major/minor band ratios (<1) indicated stable protein structures. The p35 expressing transgenic line displayed an intermediate major band ratio thus correlating with the tolerance levels observed in the salinity assays.

Another key indicator of stress is the rate of cell wall synthesis and pectin accumulation which can be observed by analysis the ester at the absorption band at 1745 cm^-1^. Studies by Yang and Yen (2002) demonstrated *Arabidopsis* plants which are sensitive to salinity treatment do not accumulate cell wall precursors. In contrast, tolerant ice plants continue to generate pectin and other cell wall precursors. As shown in **Figure 3**, cell wall synthesis in WT and transgenic controls plants was much more sensitive to salinity treatment compared to the respective transgenic lines which maintained cell wall synthesis rates. Taken together, the FTIR data clearly show that the transgenic lines contain proteins with a higher level of ordered H-bonds and continue to synthesize cell wall precursors during salinity stress than their WT and transgenic control counterparts. These results correlate with those observed in other salinity tolerant plant species such as the ice plant and suggest that the transgenic lines are able to maintain higher-ordered forms of proteins in leaves during salinity stress.

### Constitutive-Overexpression of Cell Death Regulators Enhanced Tolerance to Salinity Stress in Rice

**FIGURE 4:**
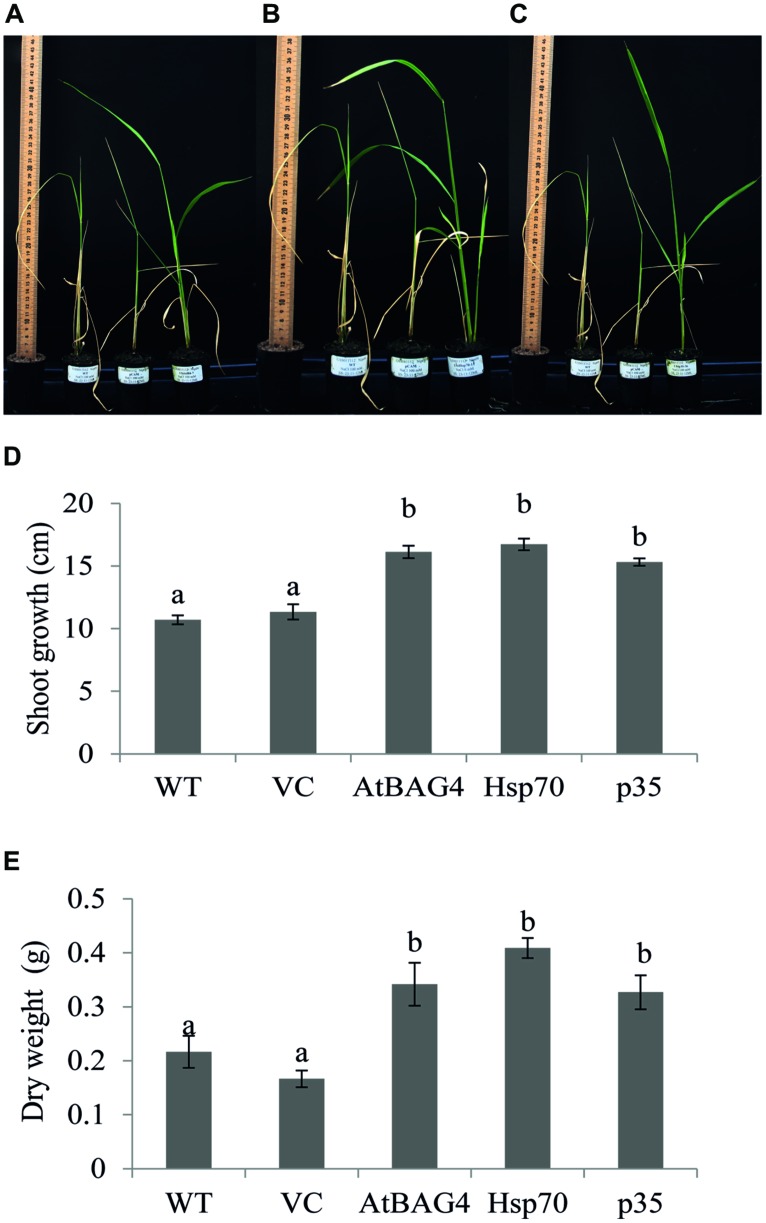
**Anti-apoptotic transgenic plants exhibit less damage and better growth than WT and VC under salinity stress at seedling stage. (A)** WT, VC and AtBAG4 plants; **(B)** WT VC and Hsp70 plants; and **(C)** WT, VC and p35 plants after 13 days exposed to 100 mMNaCl. **(D)** Shoot growth and **(E)** Dry weight of WT, VC and anti-apoptotic transgenic plants after 13 days of 100 mM NaCl exposure. Data represent the mean and SE of three replicates. Bars that share a common letter are not significantly different by Tukey HSD (Honest Significant Differences) test at 95% confidence intervals.

**FIGURE 5:**
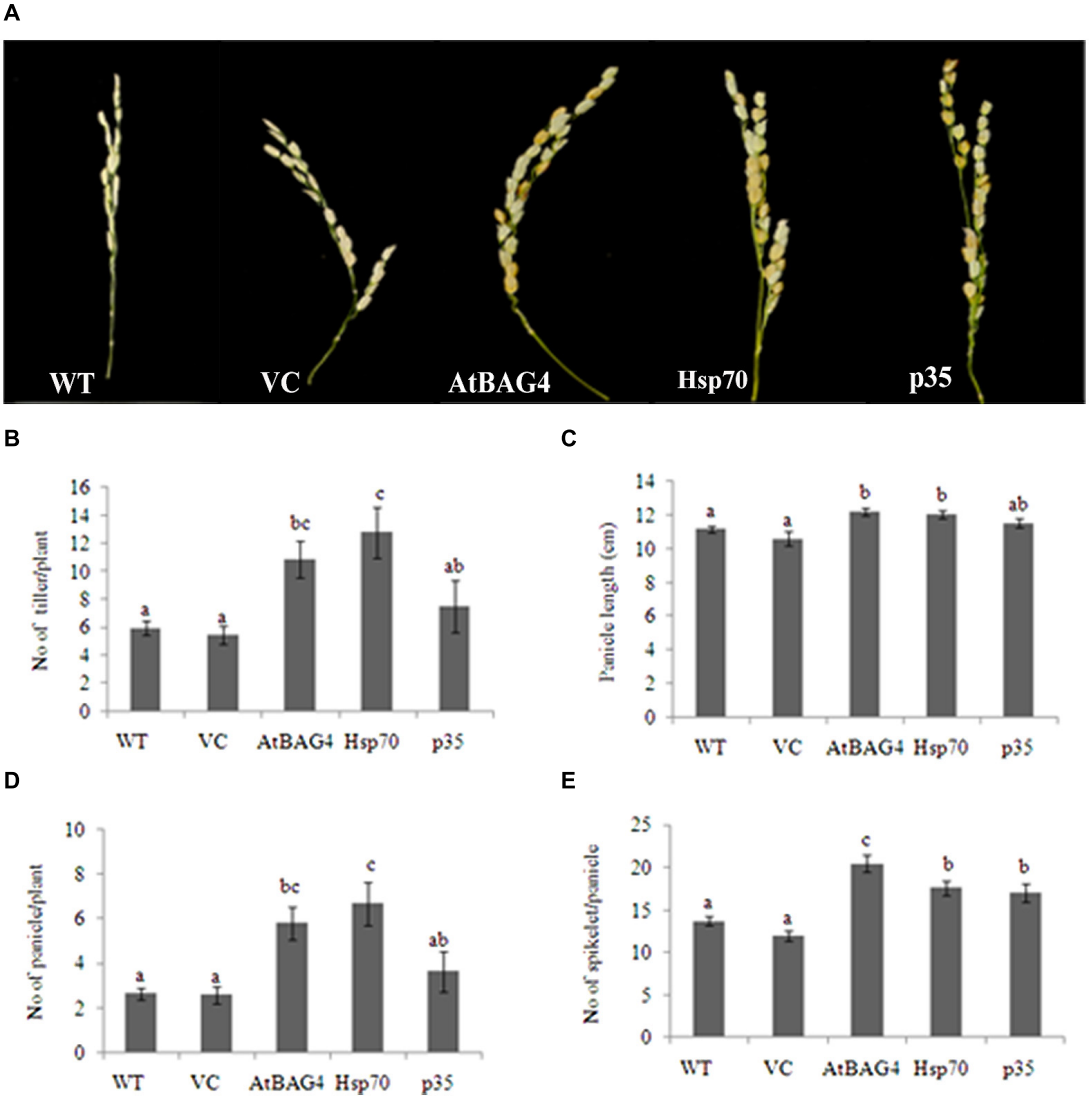
**Yield components of transgenic rice expressing *AtBAG4, Hsp70, p35* and control plants under NaCl stress condition at reproductive stage. (A)** Representative photo of panicles from WT, VC and anti-apoptotic transgenic rice. **(B)** Number of tiller per plant; **(C)** Number of panicle per plant; **(D)** Panicle length and **(E)** Number of spikelet per panicle. Data represent the mean and standard error of three replicates. Bars that share a common letter are not significantly different by Fisher’s method (Least Significant Differences) at 95% confidence intervals.

Previous reports have indicated that rice is relatively tolerant to salinity stress during germination, tillering and maturity but sensitive during seedling and reproductive stages (Heenan et al., 1988; Zeng et al., 2001). Salinity stress at seedling stage, shows the most distinct difference between shoot growth and dry weight of salt sensitive and salt tolerant cultivars (Lee et al., 2003; Cha-Um et al., 2009). Salinity stress at the reproductive stage has been reported to significantly reduce yield components, especially the number of tillers per plant, number of spikelets per panicle and panicle length (Zeng et al., 2001, 2003). These parameters therefore were examined in transgenics expressing *AtBAG4, Hsp70* and *p35* as well as VC and WT plants. Under salinity stress, shoot growth and dry weight of WT and VC plants were significantly reduced compared to that of the transgenics (**Figures 4A–E**). This result indicates that at the seedling stage, transgenic rice expressing *AtBAG4, Hsp70* and *p35* are more tolerant to salinity stress than the WT and VC plants. Salinity tolerance of the respective plants at the reproductive stage was also determined based on yield components including the number of tillers per plant, panicles length and the number of spikelet per panicle. As shown in **Figure 5**, salinity stress resulted in significantly reduced yields of VC and WT plants whereas transgenic plants expressing *AtBAG4, Hsp70* and *p35* genes exhibited significantly higher yield components.

### Transgenic Plants Expressing Anti-Apoptotic Genes Display Improved Physiological Characteristics

**FIGURE 6:**
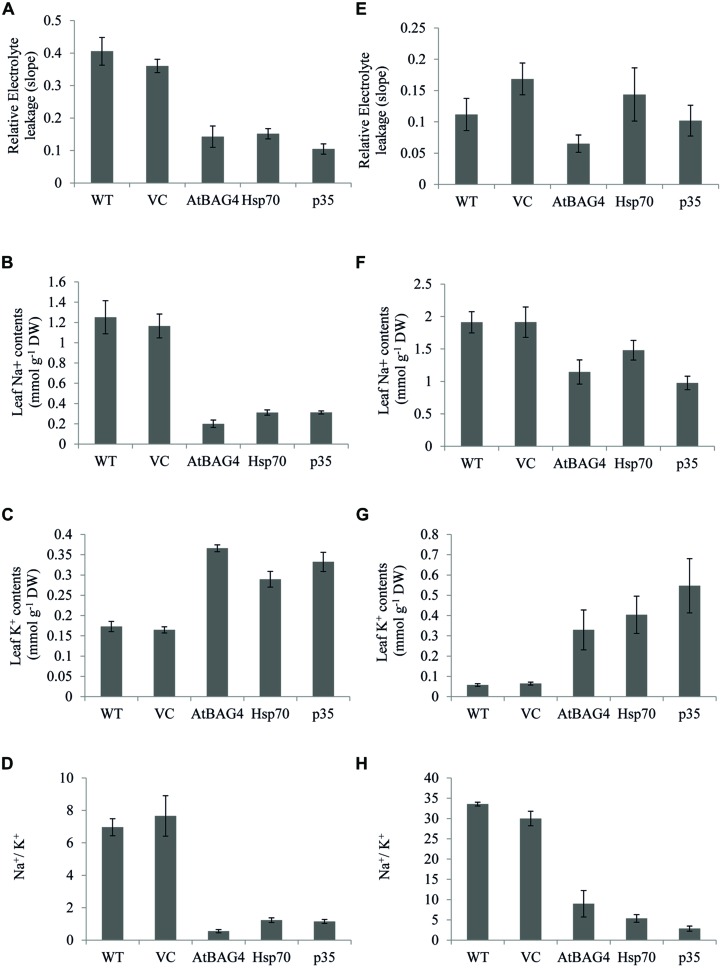
**Cell membrane integrity, Na^+^, K^+^, and Na^+^/K^+^ ratios of transgenic rice and controls under 100 mM NaCl treatment at seedling and reproductive stages. (A)** and **(E)** Relative electrolyte leakage of leaf cell at seedling and reproductive stages respectively; **(B)** and **(F)**: Na^+^ concentration in leaf at seedling and reproductive stages respectively. **(C)** and **(G)** K^+^ concentration in leaf at seedling and reproductive stages respectively. **(D)** and **(H)** Na^+^/K^+^ ratios at seedling and reproductive stages respectively. Data represent the mean and standard error of three replicates.

Membrane integrity, Na^+^, K^+^ concentrations and the ratio between Na^+^ and K^+^ are key parameters that differentiate sensitive and tolerant rice cultivars during salinity stress (Dionisio-Sese and Tobita, 1998, 2000; Cha-Um et al., 2009). Transgenics, VC and WT rice plants were exposed to 100 mM NaCl for 13 days at seedling stage and 30 days at the reproductive stage and assessed for electrolyte leakage (an indicator for membrane integrity) and leaf Na^+^, K^+^ concentrations. Results showed that relative electrolyte leakage was increased in WT and VC plants under 100 mM NaCl treatment at both the seedling and reproductive stages (**Figures 6A,D**). Electrolyte leakage was also increased in transgenic rice plants expressing anti-apoptotic genes but at lower levels when compared to WT and VC plants. These results combined with TUNEL and ROS assays indicate that transgenic rice expressing anti-apoptotic genes unlike their WT and VC counterparts can maintain cell membrane integrity during salinity stress.

Under salinity stress, tolerant rice cultivars accumulate less Na^+^ in the leaf and shoot than their sensitive counterparts (Dionisio-Sese and Tobita, 1998, 2000; Lee et al., 2003; Moradi and Ismail, 2007; Cha-Um et al., 2009; Ghosh et al., 2011). In this study, leaf Na^+^, K^+^ and Na^+^/K^+^ ratios of transgenic, VC and WT rice were assessed following exposure to NaCl stress at both the seedling and reproductive stages. As shown in **Figures 6B,E**, salinity stress results in significant accumulation of Na^+^ in WT and VC plants and subsequently an increased Na^+^/K^+^ ratio (**Figures 6G,H**). In contrast, the transgenic plants expressing *AtBAG4, Hsp70* and *p35* maintained lower Na^+^ levels and a lower Na^+^/K^+^ ratio following NaCl exposure at the seedling stage (**Figures 6C,F**). As expected, no significant differences in leaf Na^+^ content or leaf Na^+^/K^+^ ratio was observed between the transgenic plants, WT and VC plants under non-stressed conditions (**Figure S4**).

**FIGURE 7:**
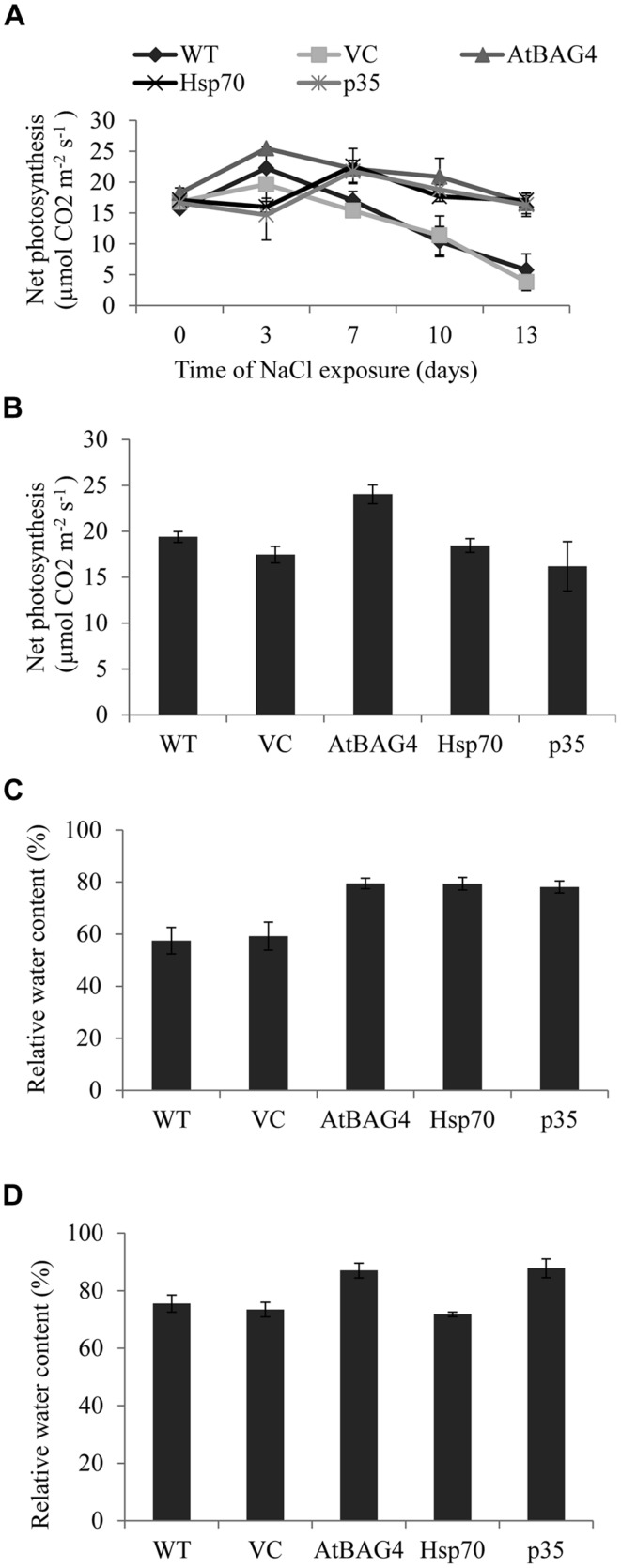
**Net photosynthesis and relative water content of transgenic rice, WT and VC under 100 mM NaCl treatment at seedling and reproductive stages. (A)** and **(B)** Net photosynthesis at seedling and reproductive stages respectively. **(C)** and **(D)** Relative water contents at seedling and reproductive stages respectively. Data represent the mean and SE of three replicates.

Photosynthesis is a fundamental physiological process that provides a vital energy source for plant growth as well as arsenal to facilitate plant adaptation to environmental and biotic stresses. During salinity stress, net photosynthesis and RWC were reported to be maintained higher in tolerant than sensitive rice cultivars (Dionisio-Sese and Tobita, 2000; Moradi and Ismail, 2007; Cha-Um et al., 2009). To investigate the potential of anti-apoptotic genes to enhance salt tolerance in rice by maintaining high photosynthesis efficiency and high RWC under salinity stress, the net photosynthesis and RWC of transgenic plants expressing *AtBAG4*, *Hsp70* and *p35* were examined and compared with WT and vector-control plants at both the seedling and reproductive stages. As shown in **Figures 7A,B**, 100 mM NaCl stress treatment resulted in differential responses between the transgenic anti-apoptotic and control plants. Although the trend of net photosynthesis was different between the plants tested in the first 7 days of salinity exposure, this difference was not statistically significant. Net photosynthesis of all transgenic and control plants decreased at day 10 in both unstressed (**Figure S4**) and salinity stressed growth conditions (**Figure 7A**). This decrease was possibly due to developmental effects such as senescence (photosynthesis was measured using the third leaf at seedling stage). However, net photosynthesis in WT and VC plants without anti-apoptotic genes subjected to salinity dropped significantly more rapidly than the transgenics. After 13 days exposure to 100 mM NaCl, net photosynthesis of WT and VC plants was significantly lower than that of the transgenic plants; and the net photosynthesis of *AtBAG4, Hsp70* and *p35* transgenic plants was not significantly different from that of their non-stressed counterparts.

The previous data showed that the anti-apoptotic transgenic rice plants can maintain cell membrane integrity during salinity stress. To determine whether this correlated with higher water retention, leaf RWCs of the transgenic, WT and VC plants in unstressed and following NaCl treatment were assessed at both the seedling and reproductive stages. As shown in **Figures 7C,D** under 100 mM NaCl treatment at the seedling stage, the transgenic plants maintained significantly higher leaf RWC compared to WT and VC plants. Leaf RWC of *AtBAG4* and *p35* transgenic rice plants following salinity treatment at the reproductive stage was significantly higher than that of the WT and VC plants (**Figure 7D**). However, no significant differences were observed between *Hsp70* transgenic plants, WT and VC plants when subjected to 100 mM NaCl at the reproductive stage (**Figure 7D**).

## Discussion

Climate change forecasts indicate that vast areas of the globe will become significantly more prone to aberrant environmental conditions. This potential crisis is exacerbated by predicted trends in population growth that suggest global food production needs to increase significantly to reach sustainable levels. The next frontier of agronomic crops should be both high yielding and tolerant to a multitude of stresses, thus enabling them to survive and yield in future environments. In this study we generated and assessed transgenic rice plants expressing the anti-apoptotic genes *AtBAG4, Hsp70* and *p35* for enhanced salinity tolerance using a variety of agronomical and physiological characteristics.

Transgenic rice expressing *AtBAG4, Hsp70* and *p35* were found to possess many characteristics that have been reported in salt tolerant rice cultivars subjected to salinity stress. This includes maintenance of ROS, growth and yield levels, minimisation of cellular membrane electrolyte leakage, high photosynthetic efficiency and low Na^+^ accumulation. In contrast, the WT and the vector-control rice plants, remained salt sensitive with serious damage at the whole plant level such as stunted growth, cell membrane damage, low photosynthetic efficiency, high sodium ion content and finally death or yield losses.

Previous studies have shown that PCD pathways are triggered by increased ROS levels, among other signals, that originate from a variety of organelles including the chloroplast and mitochondria (Foyer and Noctor, 2005, 2009; Rhoads et al., 2006). Moreover, salinity stress is associated with increased ROS accumulation that can cause significant injury and if prolonged even death (Borsani et al., 2005; Zhu et al., 2007; Chawla et al., 2013). Consistent with these reports, the level of ROS in leaves of WT, VC and transgenic rice expressing *AtBAG4, Hsp70* and *p35* increased during salinity stress. Importantly, however, despite showing increased levels, the amount of ROS observed in the transgenics was significantly lower than in the WT and VC controls. Although speculative, the genes used in this study may regulate ROS levels either directly (*p35*) or indirectly (*Hsp70* and *AtBAG4*).

Expression of *p35* has been reported to inhibit H_2_O_2_-induced PCD in insect cells by directly sequestering ROS. This antioxidant function of *p35* has been attributed to the presence of metal-binding sites in the proteins that could enhance its antioxidant property and/or its three-dimensional structure contains some amino acids that confer electro-dynamically stable configuration conducive to ROS-trapping. The antioxidant role of *p35* is also supported by the chemical radio-protectors formed by six cysteine residues in its sequence which can react with certain ROS in a constant rate (Sah et al., 1999). In agreement with this report, the results in this study show that expression of *p35* in rice is associated with lower cellular ROS levels and PCD during salinity stress.

The exact mechanistic details of how *Hsp70* and *AtBAG4* suppress cellular ROS levels remain unknown, though we can speculate on a few plausible scenarios. (i) constitutive expression of *Hsp70* and *AtBAG4* genes might reduce ROS levels indirectly by facilitating the function of cellular processes which if not working efficiently promote the generation of ROS and (ii) maintenance of ion homeostasis.

If left unchecked, copious ROS production can denature proteins. To mitigate the denaturation of proteins, cells employ a complex network of molecular chaperones and foldases which promote efficient and correct folding of cellular proteins (Hartl et al., 2011). Members of the highly conserved Hsp family are chaperones that play a key role within the promotion of correct protein folding and proteostasis control (Hartl et al., 2011). A definitive feature of the BAG family of proteins is their ability to bind and facilitate the function of HSPs (Doukhanina et al., 2006; Williams et al., 2010). The expression of *Hsp70* and *AtBAG4* may assist in the folding of proteins and prevention of protein denaturation in high ROS environments, thus maintaining efficiency of cellular processes and mitigating the production of ROS and plant damage. The suppression of ROS in transgenic plants expressing anti-apoptotic genes is perhaps one of the most important steps to protect cells from oxidative damage and enable the plants to maintain photosynthetic efficiency which in turn provides energy for plants to grow and survive under salinity stress conditions.

Accordingly, homeostasis of cellular ROS levels promotes maintenance of cellular membrane integrity. Previous studies have shown that ROS-induced cell death can result from oxidative processes such as membrane lipid peroxidation (LPO), protein oxidation (PO), enzyme inhibition and DNA, RNA damage (Mittler, 2002). The cell membrane is the first site of signal perception as well as the primary defense against abiotic stresses including salinity and it is one of the most vulnerable targets for ROS due to the predominance of lipids (Ghosh et al., 2011). Electrolyte leakage analysis in this study indicated that the cell membrane in rice expressing *AtBAG4, Hsp70* and *p35* is less damaged than in WT and VC plants during salinity stress. Cell membrane integrity probably enables the plants to maintain ion homeostasis under salinity stress. During salinity stress, increased extracellular Na^+^ concentrations create a large electrochemical gradient that favors the passive transport of Na^+^ into the cell through K^+^ transporters result in high cytosolic Na^+^ concentration (Blumwald, 2000). To maintain low cytosolic Na^+^ concentrations, plant cells need to extrude Na^+^ of the cell or compartmentalize Na^+^ into vacuoles. The main mechanism for Na^+^ extrusion in plant cells is mediated by the plasma membrane H^+^-ATPase (Sussman, 1994). As the cell membrane in WT and VC plants was damaged it could not use this strategy to pump Na^+^ out of the cell hence the Na^+^ concentration was recorded at high levels in leaf cells of those plants. On the other hand, transgenic plants expressing *AtBAG4, Hsp70* and *p35* can maintain cell membrane integrity and therefore could use the H^+^-ATPase to extrude Na^+^ thus maintaining a low concentration of Na^+^ in cytoplasm. The high maintenance of low cytosolic Na^+^ concentrations facilitates a high concentration of K^+^ therefore ensuring a low Na^+^/K^+^ ratio that could offer an optimal cellular environment for enzymes thus supporting metabolism. High cytosolic K^+^ concentrations in transgenic rice plants expressing *AtBAG4, Hsp70* and *p35* enable the plants to inhibit PCD. The maintenance of high K^+^ concentrations in cells of transgenic plants expressing anti-apoptotic genes correlated with less cell death in those plants (Hoang et al., 2014).

One of the factors that lead to yield loss in rice cultivated in salt-affected land is photosynthetic inefficiency. There is a close link between increased photosynthesis with yield (Long et al., 2006). Photosynthesis and cell growth are among the primary processes that are affected by salinity (Munns et al., 2006; Chaves et al., 2009). Under salinity stress, the net photosynthesis in sensitive rice cultivars was significantly decreased in comparison to that in tolerant cultivars (Dionisio-Sese and Tobita, 2000; Moradi and Ismail, 2007; Cha-Um et al., 2009). Previous studies have suggested that a low Na^+^/K^+^ ratio improves photosynthesis and overall plant growth (Rodrigues et al., 2013). The maintenance of photosynthetic capacity in Barley, wheat and rice under salinity stress was associated with low Na^+^, high K^+^ and a low Na^+^/K^+^ ratio in cytoplasm (James et al., 2006). Consistent with these reports, transgenic plants expressing *AtBAG4, Hsp70* and *p35* maintained low cytosolic Na^+^, high K^+^ and a low Na^+^/K^+^ ratio which promoted high photosynthetic capacity. Maintaining high photosynthetic efficiency provides essential energy/additional artillery for transgenic plants to cope with salinity stress as energy is required for many cellular processes to sustain growth; energy is also required for pumping the Na^+^ out of cells and supports reduced levels of ROS. It is evident that reduced photosynthetic rates increase the formation of ROS (Apel and Hirt, 2004; Foyer and Noctor, 2005; Foyer and Shigeoka, 2011).

Transgenic rice expressing *AtBAG4, Hsp70* and *p35* maintain growth rate (shoot growth and dry weight) and yield components (number of panicles per plant and number of spikelets per panicle) during salinity stress. This is probably also a result of the maintenance of high cytosolic K^+^ and homeostasis in transgenic rice plants expressing *AtBAG4, Hsp70* and *p35*. In response to osmotic stress caused by salinity, shoot growth rate decreases immediately (Munns and Tester, 2008). High cytosolic K^+^ in transgenic plants expressing anti-apoptotic genes allow plants to adjust to osmotic stress and maintain high growth rates (Maathuis and Amtmann, 1999). The maintenance of growth rate leads to higher yield components in transgenic rice expressing *AtBAG4, Hsp70* and *p35* in comparison to WT and VC which had very low cytosolic K^+^ under salinity stress condition. Another factor that causes reduced growth rates in high salt environments is inadequate photosynthesis due to limited carbon dioxide uptake as a consequence of stomatal closure (Zhu, 2001). *AtBAG4, Hsp70* and *p35* transgenic rice plants maintained high net photosynthesis which provided ample energy for their growth and development.

The three genes assessed in this study all of different functions at the cellular level, but are all involved in the suppression of PCD. The decision of whether a given cell lives or dies is obviously an extremely important one for the wellbeing of the organism and hence PCD pathways are regulated by a series of checks or balances which are dictated by the pro- and anti-survival machinery. The master switch of PCD pathways and the cell life/death decision during salinity as well as other stresses is the balance of the pro-death and anti-apoptotic signals within that given system. Expression of anti-apoptotic genes coincided with reduced pro-death signals such as ROS levels which in turn supported the maintenance of cell membrane integrity and Na^+^ homeostasis. Low ROS levels reduce the risk of cellular damage caused by LPO and PO; two established hallmarks of oxidative damage. The maintenance of membrane integrity and Na^+^ homeostasis promoted sustained photosynthetic efficiency which in turn provided energy for growth. Maintenance of ROS levels also facilitates photosynthesis and growth by minimizing LPO in chloroplasts [see (Foyer and Shigeoka, 2011; Considine and Foyer, 2014; Noctor et al., 2014)]. Well-maintained growth further dilutes Na^+^ concentration which helps maintain Na^+^ homeostasis leading to the increased membrane integrity, RWC, net photosynthesis and finally growth and yield. Hence the transgenic rice was able to minimize the toxicity caused by the accumulation of Na^+^ and water deficit under salinity stress.

In conclusion this research focused on the improvement of salinity tolerance in rice by manipulating PCD pathways. The exogenous expression of three anti-apoptotic genes from different sources in this study displayed similar physiological and biochemical characteristics during salinity treatment thus providing further evidence that cell death pathways are conserved across broad evolutionary kingdoms. This study also provided mechanistic evidence for the biochemical and physiological basis of salinity tolerance in transgenic rice expressing anti-apoptotic genes and proposes that anti-apoptotic genes improve stress tolerance by “creating” an optimal cellular environment that minimizes pro-death signals including accumulation of ROS. Once created, this environment facilitates cellular metabolism even during stressful conditions to suppress flicking of the “cellular kill switch” that is apoptotic-like cell death.

## Conflict of Interest Statement

The authors declare that the research was conducted in the absence of any commercial or financial relationships that could be construed as a potential conflict of interest.
