# Supplementary material for: Development of salinity tolerance in rice by constitutive-overexpression of genes involved in the regulation of programmed cell death
Source: Front Plant Sci. 2015 Mar 30;6:175. doi: 10.3389/fpls.2015.00175 (PMC4378369; doi:10.3389/fpls.2015.00175)
Supplement: Supplementary file 1 [file data_sheet_1.pdf]

Supplementary data

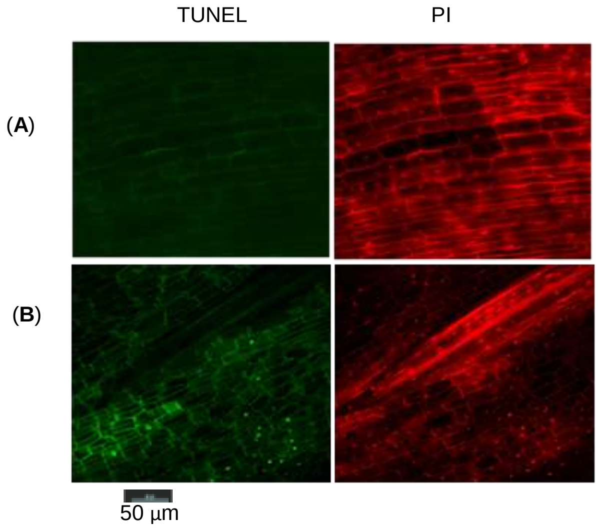

Figure 1: Salinity induces cell death in wild type *Oryza sativa* L. ssp Japonica cv. Nipponbare. (**A**) Root tip of Wild type plants grown in normal condition. (**B**) Root tip of wild type plants grown in stress condition (100 mMNaCl added). TUNEL assay and propidium iodide counter-staining were conducted at 36h after salinity stress. Nucleic acid in TUNEL positive cells is selectively stained and fluoresces green, indicating the presence of apoptotic-like bodies, whereas all nucleic acid is counter-stained with propidium iodide and fluoresces red. Magnifications as indicated.

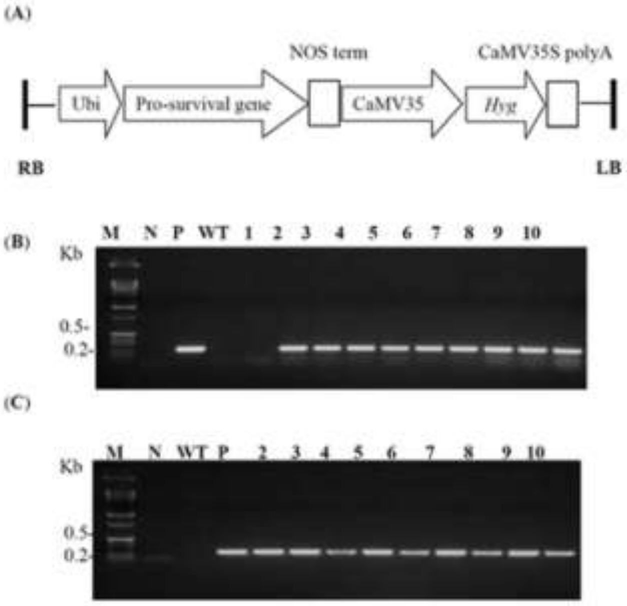

Figure 2: Schematic diagram of gene construct and molecular analysis of transgenic lines expressing pro-survival genes. (**A**) Schematic diagram of pro-survival overexpression gene construct. LB, left border RB, right border. (**B**) Example of PCR confirmation of transgenic lines; M, marker; N, negative control; Pov, positive control (plasmid DNA); 1-10, *AtBAG4* independent trangenic lines 1-10. (**C**) Example of RT-PCR analysis on transgenic plant expressing *AtBAG4*

Table 1: Confirmation of transgenics and gene expression

Gene of interest	PCR positive (# lines)	RT-PCR positive (# lines)	(% RT-PCR positive)
AtBAG4	9	9	100
Hsp70	15	15	100
p35	32	29	92.8
Total	56	53	94.6

Table 2: primers used for molecular characterization of transgenic rice

Gene	Sequences	Tm	PCR product length (bp)
AtBAG4	5'TGTGGATTTTTTTAGCCCTGCCTTC 3'<br>5'CAAAAAGCGGCGGAAGCAGAGTC 3'	65<br>66	220
Hsp70	5'TGTGGATTTTTTTAGCCCTGCCTTC 3'<br>5'ACCAAAGTCTAAACCCAGAAGCACC3'	65<br>66	110
p35	5'TAGAGTTTACTACAGAATCGAGCTGG 3'<br>5'GTAAAACGACGGCCAGT 3'	65<br>55	450
Vector control(hpt)	5'AGC TGC GCCGATGGT TTC TAC AA3'<br>5'ATC GCCTCG CTC CAGTCA ATG 3'	62<br>59	700
VirC	5'GCCTTAAAATCATTTGTAGCGACTTCG 3'<br>5'TCATCGCTAGCTCAAACCTGCTTTCTG 3'	62<br>62	738

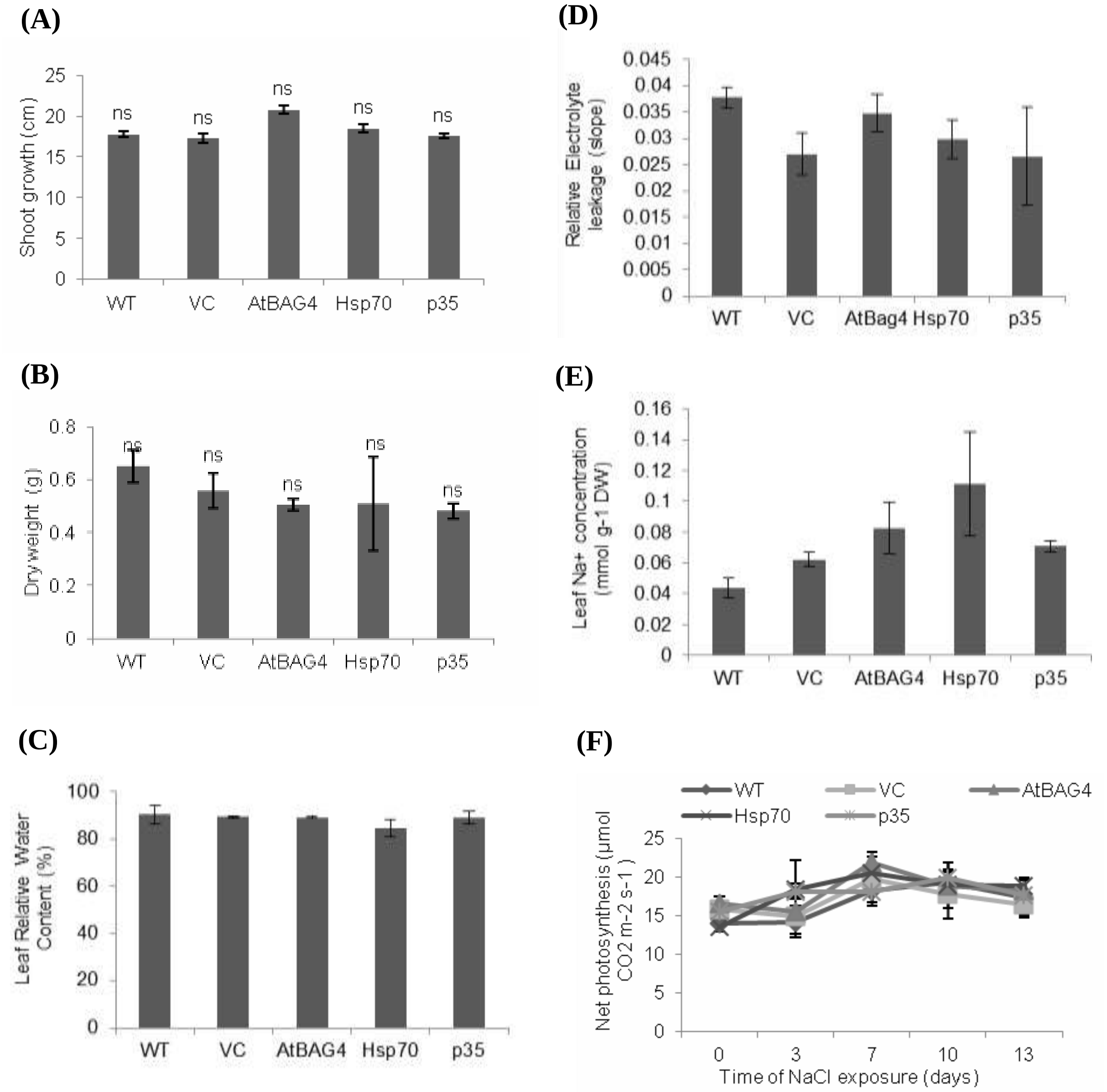

Figure 3: Morphological and physiological data of transgenic rice expressing *AtBAG4*,*Hsp70* and *p35*,VC and WT under normal growth condition at seedling stage. Data represent the mean and standard error of three replicates.

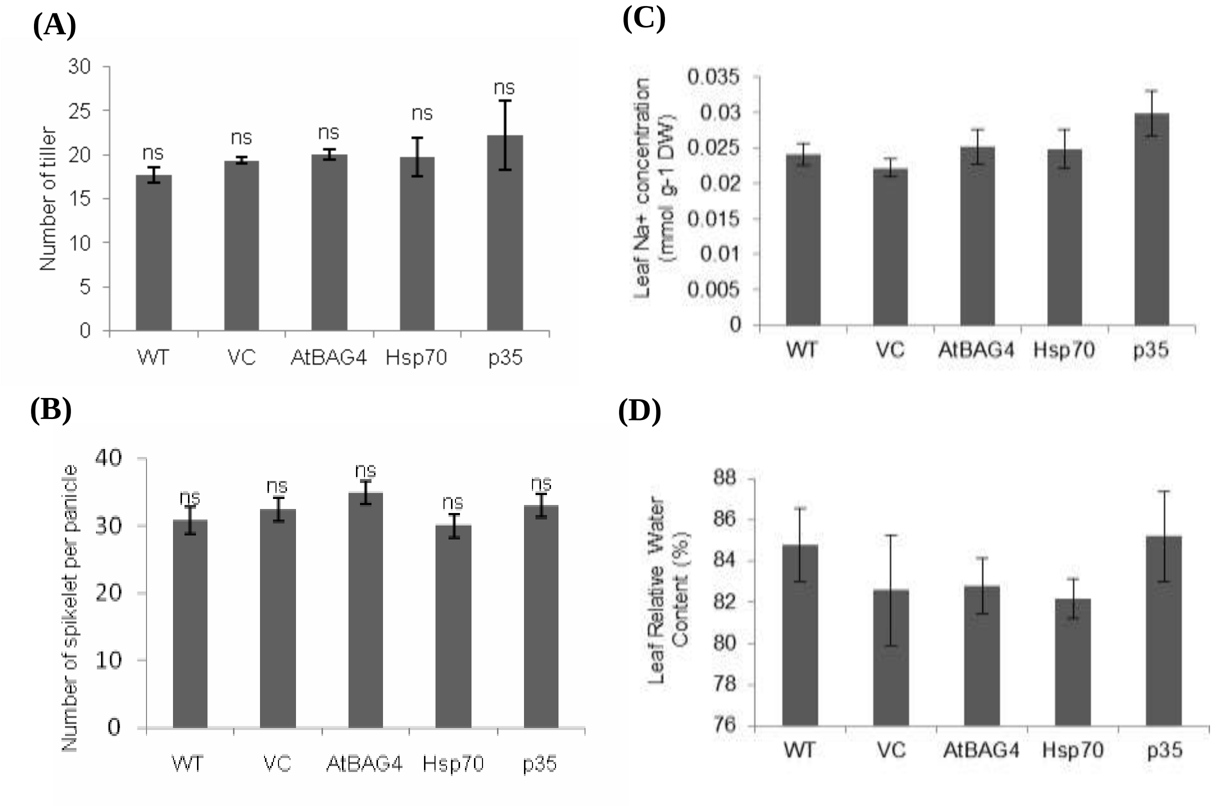

Figure 4: Morphological and physiological data of transgenic rice expressing *AtBAG4*,*Hsp70* and *p35*,VC and WT under normal growth condition at reproductive stage. Data represent the mean and standard error of three replicates.

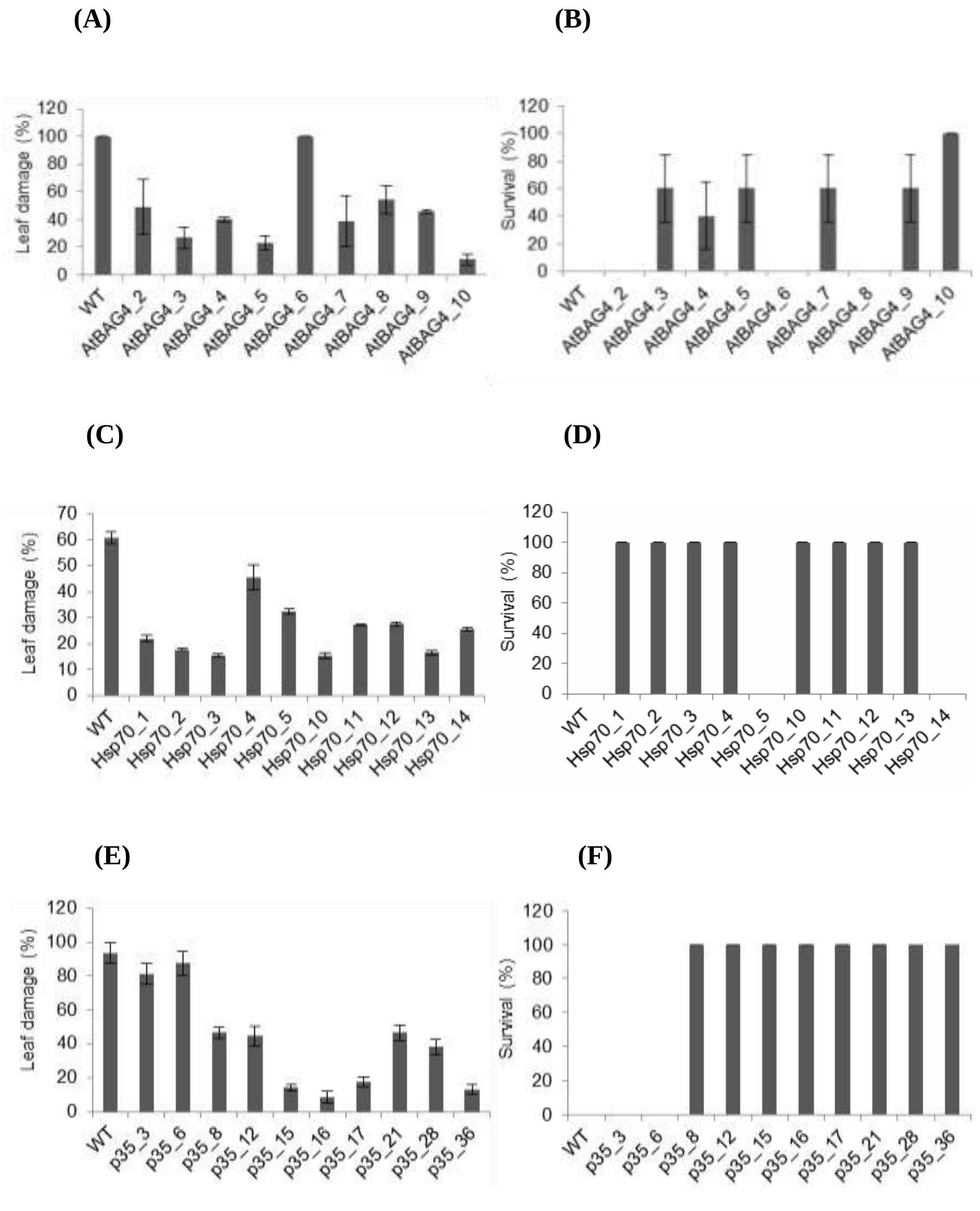

Figure 5: High throughput screening for salinity tolerance in transgenic rice lines expressing anti-apoptotic genes at seedling stage based on leaf damage and survival rate. 10 transgenic lines expressing each of the anti-apoptotic genes were subjected to 100 mM NaCl for 3 weeks. Leaf damage was assessed at day13 and survival rate was assessed at day 21post-salinity stress. (A)&(B) Leaf damage and survival rate of *AtBAG4* transgenic rice plants. (C)&(D) Leaf damage and survival rate of *Hsp70* transgenic rice plants. (E)&(F) Leaf damage and survival rate of *p35* transgenic rice plants.
